# The Royal Navy Medical Service

**Published:** 1911-09-09

**Authors:** 


### SPECIAL ARTICLE.

### THE ROYAL NAVY MEDICAL SERVICE.

### WHAT THE NEW REGULATIONS MEAN.

To Admiral Sir John Durnford, ably aided by the Director-General Sir James Porter (in the future Surgeon-General) and the Committee of Experts that has sat at the Admiralty for so long, the senior service is indebted for what promises to be one of the most perfect organisations for post-graduate study that has yet been devised.

#### The Naval Surgeon of To-day.

The naval surgeon will for the first time be put on as good a footing as his colleague under the War Office or the India Office, and he will be afforded instruction that will equip him to efficiently discharge the important and often onerous duties that devolve upon him in peace and in war. It must be borne in mind (and this fact was no doubt recognised by the Committee) that the naval surgeon serves under exceptional conditions; he is as a rule more isolated in emergency than his colleagues in the services of the War Office or India Office or those in private practice, and it is essential above all things that he shall have at reasonably frequent intervals the opportunity to study the latest developments of his profession. The new school at Greenwich will supply this in a very efficient manner.

#### Teachers of the School.

That part of the scheme which will be most acceptable to the service is doubtless the confidence that is to be reposed in those officers who have attained distinction in their profession and shown an aptitude to teach. A feature of the school will be that officers in the service will be selected as Professors, especially in the departments of bacteriology, pathology, and hygiene, but those who are selected for study will also have the opportunity and advantage of studying under some of the greatest teachers of the day. The junior men will be required to attend definite hospital cliniques, but senior men will be afforded an opportunity to specialise and seek their information in a wider area. There are men in the Navy, well known, who have shown their aptitude as teachers, and these we may expect to see at Greenwich.

#### The Dreadnought Hospital.

The Dreadnought Hospital will doubtless supply the greater amount of clinical material not only for clinical lectures, but also to provide matter for the new laboratories. For some years naval surgeons have been seconded for study at the Dreadnought Hospital by being made students at the London School of Clinical Medicine attached to that hospital, and the lectures and demonstrations in this school will be utilised especially for the junior men. In addition to this, senior and selected men will be given an opportunity to attend a three months' course at the London School of Tropical Medicine, while others will be allowed to specialise at the various teaching centres in London.

#### Post-Graduate Teaching.

The admirable scheme evolved by the Committee will not only improve the service by giving greater opportunities for study and attracting thereby a better class of graduates, but the all-important question of pay and emoluments has secured very careful consideration. Increased increments of pay will be more frequent and promotion much accelerated. In part this will be effected by a judicious rejection to retirement of those who fail to make themselves efficient by means of the instruction afforded or who fail to pass the prescribed examinations which will be held at the expiration of each study leave. The titles of the senior officers have been altered, and with the abolition of the cumbrous title of Inspector-General of Hospitals and Fleets will be revived the time-honoured designation of Surgeon-General, a post that the best men in the medical profession may be proud to attain to.

#### The Future of the Service.

Altogether the new scheme will vastly improve the medical service of the Navy, and will attract to it better men. There have been those who have decried the Navy as a career for the medical graduate. It has been suggested that the surgeon should be engaged for the commission as the surgeon is engaged for the voyage in the merchant service, but the action of the Admiralty is a satisfactory reply to such criticism. The medical service of the Navy under the new regulations is likely to attract to it medical men of the highest attainments, for it opens up prospects which the best may aspire to attain to. The naval surgeon has always been a *persona grata* among his colleagues in the ward-room, and the improvement in his status both as an officer and a medical man is not likely to lessen his popularity in the future.

